# Stercoral colitis mimicking appendicitis

**DOI:** 10.1186/s12245-017-0134-y

**Published:** 2017-02-20

**Authors:** Abdelghafour Elkoundi, Mustapha Bensghir, Charki Haimeur

**Affiliations:** Department of Anesthesiology and Intensive Care, Military Hospital Mohammed V, Faculty of Medicine and Pharmacy of Rabat, Mohammed V University, Rabat, Morocco

**Keywords:** Fecal impaction, Stercoral colitis, Appendicitis, CT scan

## Abstract

**Background:**

Stercoral colitis is an inflammatory process involving the colonic wall related to fecal impaction. This rare condition is associated with high morbidity-mortality.

**Findings:**

We report a case of a 78-year-old woman with a history of dementia under clozapine who presented a clinical and sonographic presentation of acute appendicitis. The worsening of her clinical condition prompted us to review our diagnosis and modify our approach using the CT scan which was consistent with stercoral colitis. This report concerns an atypical presentation of this condition.

**Conclusions:**

The present case highlights the ability of severe forms of fecal impaction to precipitate very rare and life-threatening complications like stercoral colitis. It also points the importance of including stercoral colitis in the differential diagnosis of acute appendicitis in altered patients under anticholenergic drugs and the critical role of the CT scan as a crucial radiologic adjunct.

## Findings

### Case synopsis

A 78-year-old woman with a history of dementia, depression, and hypertension presented to the emergency department with a 3-day history of lethargy and confusion. Among the medications she was taking were clozapine for over 10 years and amlodipine. On examination, she was drowsy, tachycardic (117 beats per minute), and hypotensive (82/42 mmHg) with a temperature of 38 °C. The abdomen was distended with right lower quadrant abdominal pain and active bowel sounds. Rectal examination disclosed a small amount of stool. Blood tests showed raised inflammatory markers with a C-reactive protein of 320 mg/L and a white cell count of 18 × 10^9^/L with normal urea, electrolytes, and liver function tests. Blood gasses showed metabolic acidosis with lactic acid of 4.9.

Ultrasonography abdomen revealed dilated bowel loops with a dilated appendix in a retrocecal position. Broad-spectrum empiric antibiotics were started, and the surgeon was contacted to perform appendectomy.

**Fig. 1:**
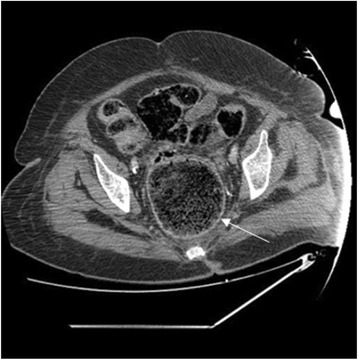
Pelvic CT scan showing a huge fecal impaction with thickened and edematous rectal wall (*arrow*)

**Fig. 2:**
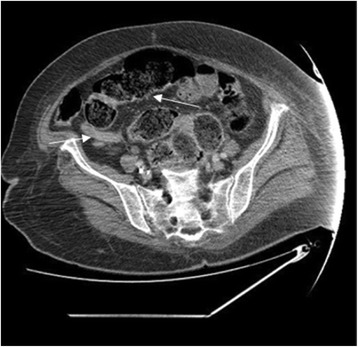
Abdominal CT scan showing fecal impaction in dilated ascending and transverse colon associated with retrocecal appendicitis and pericolic fat stranding (*arrows*)

Nevertheless, acute respiratory distress with shock occurred rapidly requiring initiation of mechanical ventilation and the use of vasoactive agents. Blood cultures were obtained, which subsequently grew *Escherichia coli* susceptible to previous antibiotics. With this recent event, the diagnosis of acute appendicitis as the cause of septic shock seemed very unlikely. A CT scan of the abdomen and pelvis was then performed to clarify the diagnosis that showed thickened and edematous rectal wall (Fig. 1), impacted stool throughout a markedly dilated rectosigmoid colon associated with retrocecal appendicitis and pericolic fat stranding (Fig. 2). The patient was taken to the operating room for an urgent exploratory laparotomy and found to have clear fluid in the peritoneal cavity without any obvious perforation. We noted inflammatory changes in the wall of the appendix, rectum, and sigmoid colon, consistent with the diagnosis of stercoral colitis. Appendectomy was performed, and the proximal colon and rectal stump were manually disimpacted with massive peritoneal lavage. Unfortunately, despite all appropriate therapy efforts, she continued to deteriorate and subsequently succumbed 3 days later.

### Stercoral colitis

Fecal impaction (FI) is a common gastrointestinal disorder with potential for major morbidity, especially in the elderly population. Risk factors for this condition include cognitive impairment, cerebral palsy, immobility, rectal hyposensitivity, poor water intake, and use of constipating drugs. Constipation can be a serious side effect of clozapine [1]. The mechanism is most likely the anticholinergic and antiserotonergic effects of the drug.

FI causes increased colonic intraluminal pressure that exceeds the capillary perfusion pressure in the bowel wall [2] and results in intestinal suffering, an uncommon condition called stercoral colitis. There is a high degree of mortality associated with this disease ranging from 32 to 57% [3]. If left untreated, it may result in a variety of complications, including perforation, peritonitis, and sepsis secondary to bacteremia and absorption of toxins into the blood stream.

CT scan is the modality of choice for diagnosis and shows a thickened rectum impacted with feces, dilated rectosigmoid colon with pericolic fat stranding [4]. In the present case, stercoral colitis mimicked appendicitis in both clinical and sonographic findings. CT scan rectified the diagnosis, which had a great implication regarding the choice of the surgical approach. Management involves prevention of constipation, aggressive fecal disimpaction, and occasionally surgical intervention.

The present case highlights the ability of severe forms of FI to precipitate very rare and life-threatening complications like stercoral colitis. It also points the importance of including stercoral colitis in the differential diagnosis of acute appendicitis in altered patients under anticholenergic drugs and the critical role of the CT scan as a crucial radiologic adjunct.

## Abbreviations

CT: Computed tomography
FI: Fecal impaction
